# *TGFBI* gene mutation analysis in a Chinese pedigree of Reis-Bücklers corneal dystrophy

**Published:** 2010-03-31

**Authors:** Ke Ma, Guo Liu, Yin Yang, Man Yu, Ruifang Sui, Wenhan Yu, Xiaoming Chen, Yinping Deng, Naihong Yan, Guiqun Cao, Xuyang Liu

**Affiliations:** 1 Ophthalmic Laboratories & Department of Ophthalmology, West China Hospital, Sichuan University, Chengdu, P.R. China; 2 Department of Ophthalmology, Peking Union Medical College Hospital, Peking Union Medical School, Chinese Academy of Medical Sciences, Beijing, P.R. China

## Abstract

**Purpose:**

To analyze transforming growth factor beta-induced (*TGFBI*) gene mutations in a Chinese pedigree with Reis-Bücklers dystrophy (RBCD).

**Methods:**

In a four-generation Chinese family with Reis-Bücklers dystrophy, six members were patients and the rest were unaffected. All members of the family underwent complete ophthalmologic examinations. Exons of *TGFBI* were amplified by polymerase chain reaction, sequenced, and compared with a reference database. The sequencing results were reconfirmed by polymerase chain reaction-restriction fragment length polymorphism (PCR-RFLP).

**Results:**

A single heterozygous C>T (R124C) point mutation was found in exon 4 of *TGFBI* in all six members of the pedigree affected with RBCD, but not in the unaffected members.

**Conclusions:**

Within this pedigree, RBCD segregates with the R124C variance, which is a known mutation for lattice corneal dystrophy type I. Therefore, along with G623D and R124L, the R124C mutation in *TGFBI* is also found to be responsible for RBCD.

## Introduction

Inherited corneal dystrophy is mainly classified as lattice, granular, Avellino, Thiel-Behnke, and Reis-Bücklers corneal dystrophy (RBCD/CDB1, OMIM 608470). RBCD, a dominantly inherited dystrophy, is characterized by bilateral, progressive, and painful corneal erosions, and significant visual impairment [1]. Histopathologically, the involvement of the Bowman’s layer, the presence of band-shaped granular and subepithelial deposits that stained intensely red with Masson trichrome, and “rod shaped bodies” in cornea were confirmed in this disease [2,3]. The mechanisms remain unclear, but it is generally accepted that transforming growth factor beta-induced (*TGFBI*) is actively involved in the pathogenesis of RBCD.

Initially known as kerato-epithelin, *TGFBI* is an extracellular matrix protein induced by transforming growth factor-beta 1 and is highly expressed in the corneal epithelium. It contains a Arg-Gly-Asp (RGD) motif that acts as a ligand recognition sequence for several integrins, and thus is associated with cell-collagen interactions with a role in the regulation of cell-adhesion. Therefore, it is believed that *TGFBI* plays a role in corneal development and wound healing by mediating cell adhesion via its interaction with collagen, fibronectin, and integrins. The human *TGFBI* gene encodes a 682 amino acids protein (68 kDa) with four internal homologous repeats. Currently, more than 30 mutations in *TGFBI* have been demonstrated in four different types of corneal dystrophies [4].

Mutated *TGFBI* has been linked to the four types of corneal dystrophy, including RBCD [5-11]. Two mutations, R124L [12,13] and G623D [14], have previously been identified in patients with typical RBCD, including Chinese RBCD pedigrees. In this study, R124C mutation, rather than R124L or G623D, was identified— to the best of our knowledge—for the first time in a Chinese family with RBCD.

## Methods

### Patient recruitment

**Figure 1:**
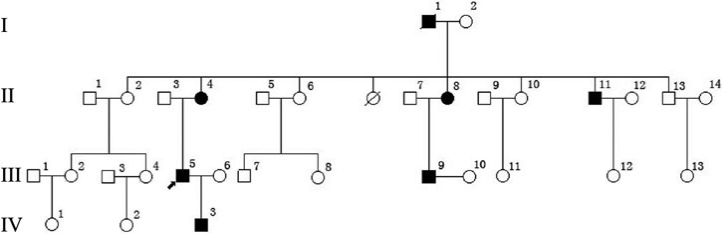
The pedigree of the family with Reis-Bücklers dystrophy (RBCD). The circle indicates female, the square indicates male, and the filled circle or square indicates the affected individual with RBCD. Arrow signifies the proband and slash through symbol indicates death.

A four-generation Chinese family from the Sichuan province with RBCD was included in this study (Figure 1). This study includes six RBCD patients and six unaffected relatives. This study was approved by a local institutional medical ethics committee, and informed consent conforming to the tenets of the Declaration of Helsinki was obtained from each of participants.

### Clinical examination

A Snellen best-corrected visual acuity test, a slit-lamp biomicroscopy, and a fundus examination were conducted by an experienced ophthalmologist for all subjects. Laser scanning in vivo confocal microscopy (Heidelberg Retina Tomograph III, Rostock Corneal Module [RCM]; Heidelberg Engineering GmbH, Heidelberg, Germany) were also performed on some of the affected and unaffected individuals.

### DNA extraction and polymerase chain reaction

Peripheral blood samples were drawn from six RBCD patients and six unaffected members. Leukocyte DNA was extracted from 200 μl peripheral blood using a TIANamp Genomic DNA Kit (Tiangen Biotech Co. Ltd, Beijing, China), following the manufacturer’s instructions. DNA integrity was evaluated by 1% agarose gel electrophoresis. The intronic primers flanking the exons were designed based on genomic sequences of *TGFBI* (Consortium Human Build 37 NC_000005) and synthesized by Invitrogen Company (Carlsbad, CA). The sequences of the primers are listed in Table 1.

**Table 1:** Primers used in polymerase chain reaction for amplification of *TGFBI*.

**Exon**	**Primer direction**	**Sequence (5′→3′)**	**Annealing temperature (°C)**
1	Forward:	GCTTGCCCGTCGGTCGCTA	62
	Reverse:	TCCGAGCCCCGACTACCTGA	
2	Forward:	AGGCAAACACGATGGGAGTCA	60
	Reverse:	TAGCACGCAGGTCCCAGACA	
3	Forward:	CCAGATGACCTGTGAGGAACAGTGA	60
	Reverse:	CCTTTTATGTGGGTACTCCTCTCT	
4	Forward:	TCCTCGTCCTCTCCACCTGT	58
	Reverse:	CTCCCATTCATCATGCCCAC	
5 and 6	Forward:	CCTGGGCTCACGAGGGCTGAGAACAT	64
	Reverse:	GCCCCTCTTGGGAGGCAATGTGTCCC	
7	Forward:	GTGAGCTTGGGTTTGGCTTC	63
	Reverse:	ACCTCATGGCAGGTGGTATG	
8	Forward:	TGAGGTTATCGTGGAGTG	53
	Reverse:	CACATCAGTCTGGTCACA	
9	Forward:	ACTCACGAGATGACATTCCT	60
	Reverse:	TCCAGGGACAATCTAACAGG	
10	Forward:	TAGAAGATACCAGATGTTAAGG	56
	Reverse:	TGTCAGCAACCAGTTCTCAT	
11	Forward:	CCTGCTACATGCTCTGAACAA	58
	Reverse:	GAATCCCCAAGGTAGAAGAAAG	
12	Forward:	GACTCTACTATCCTCAGTGGTG	58
	Reverse:	ATGTGCCAACTGTTTGCTGCT	
13	Forward:	CATTAGACAGATTGTGGGTCA	60
	Reverse:	GGGCTGCAACTTGAAGGTT	
14	Forward:	GCGACAAGATTGAAACTCCAT	58
	Reverse:	CTCTCCACCAACTGCCACAT	
15	Forward:	CCCTCAGTCACGGTTGTT	58
	Reverse:	GGAGTTGCCTTGGTTCTT	
16	Forward:	CTTGCACAACTTATGTCTGC	58
	Reverse:	TGCACCATGATGTTCTTATC	
17	Forward:	AGTGAAGTTTCACAAACCAC	58
	Reverse:	CCACATTTGGGATAGGTC	

Summary of the primers and annealing temperatures used for the amplification of the 17 exons of *TGFBI*.

DNA fragments were amplified by PCR using a MyCycler thermocycler (Bio-Rad, Hercules, CA). The 30 μl PCR reaction mixture included 1× PCR buffer (GC buffer I were used for several reactions, see Table 1), 30 ng DNA, 2.5 mM MgCl_2_, 0.3 mM of each of dNTPs, 1.5U Pfu DNA polymerase, and 1.0 μM of each of the forward and reverse primers. All reagents used in this procedure were purchased from TaKaRa Biotechnology Co., Ltd. (Dalian, China). The reactions were incubated at 94 °C for 3 min, followed by 35 cycles of 94 °C for 10 s, 53–64 °C for 30 s, and 72 ° for 1–2 min, followed by a final extension at 72 °C for 5 min.

### Sequencing and PCR-RFLP analysis

The PCR products were purified with a TIANgel Midi Purification Kit (Tiangen Biltech Co. Ltd, Beijing, China) and sequenced using an ABI 377XL automated DNA sequencer (Applied Biosystems, Foster City, CA). Sequence data were compared pair-wise with the published *TGFB1* sequences. To confirm the results of sequence analysis, a commonly used method of polymerase chain reaction-restriction fragment length polymorphism analysis (PCR-RFLP) [15] was performed, as the pathogenic mutation (R124C) resulted in a deprivation of a restriction endonuclease AciI recognition site. Exon 4 of *TGFBI* was amplified from each family member by PCR using the primers mentioned above, purified by a PCR purification kit (Qiagen, Hamburg, Germany) and digested with an AciI restriction enzyme (New England Biolabs, Inc., Beverly, MA) for 3 h at 37 °C. Fragments were analyzed by electrophoresis.

## Results

### Clinical presentation

**Figure 2:**
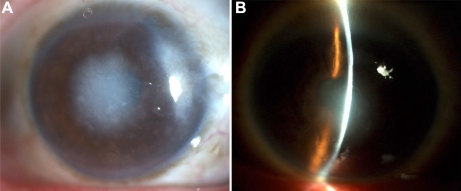
Slit-lamp examination showed an irregular corneal surface and several discrete, gray-white opacities in the subepithelial area and Bowman’s layer. Some of the opacities were associated with marked corneal scarring. No neovascularization and stromal lattice were observed.

In the family, six individuals with RBCD and six unaffected individuals were examined. The proband (a 33-year-old male, patient III:3, Figure 1) experienced recurrent photophobia, progressive vision loss, and corneal erosion since the age of 10. At the time of the examination, the best corrected visual acuity was hand motion (OD) and 6/600 (OS). Slit lamp examination showed multiple annular grayish opacities at the subepithelial and anterior stroma of the central cornea of both eyes. Representative clinical photographs of the cornea of an affected family member are shown in Figure 2. Patient IV:3, the 4-year-old son of the proband, presented with no clinical symptoms, but manifested bilateral diffuse and small dot epithelial and subepithelial opacities in the central cornea **(**Figure 2).

**Figure 3:**
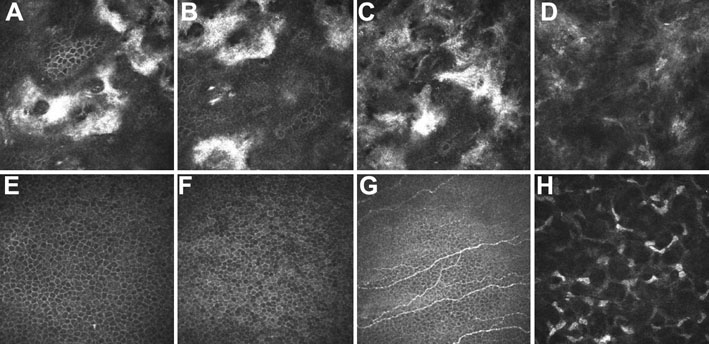
In vivo laser scanning confocal microscopy. **A**–**D**: Patient III:3 36 μm (**A**), 42 μm (**B**), 59 μm (**C**), and 110 μm (**D**) from the corneal surface. Focal deposition of homogeneous reflective materials with rounded and hyporeflective edges were observed (**A**–**C**). Amyloid-like deposits that were hyperreflective and with poorly demarcated margins were observed (**D**). **E**–**H**: Normal individual: 36 μm (**A**), 42 μm (**B**), 57 μm (**C**), and 113 μm (**D**) from the corneal surface. Epithelium cells (**E**, **F**) nerve fibers (**G**), and corneal stromal cells (**H**) were observed.

In vivo laser scanning confocal microscopy was performed on the proband. Focal depositions of homogeneous reflective materials with rounded and hyporeflective edges were observed **(**Figure 3).

### *TGFBI* mutation analysis

**Figure 4:**
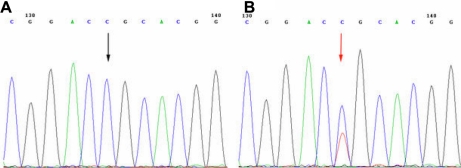
*TGFBI* heterozygous mutation in the family. **A**: Unaffected individuals of the family. **B**: Patients. The sequence in an affected member shows a heterozygous C>T transversion (indicated by the arrow).

**Figure 5:**
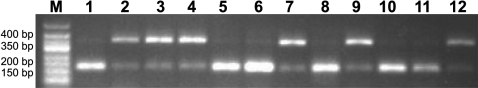
PCR-RFLP. Analysis of R124C mutation by polymerase chain reaction restriction fragment length polymorphism (PCR-RFLP). Lane 1–12: II:2, II:4, II:8, II:11, II:13, III:4, III:5, III:6, III:9, III:13, IV:1, and IV:3 of the pedigree. Lane 2, 3, 4, 7, 9, and 12 are affected individuals, and the rest are unaffected individuals of the pedigree.

A single heterozygous C>T mutation was found (R124C) in exon 4 of *TGFBI* in all affected members of this pedigree (Figure 4). This R124C mutation co-segregated with the disorder within the family. This mutation causes an Arginine to Cysteine substitution at the protein level. The results of the sequencing analysis were confirmed by PCR-RFLP analysis (Figure 5). After digestion, wild type alleles were cut into two fragments, 181 bp and 172 bp, whereas the affected patients’ alleles were cut into three fragments, 353 bp, 181 bp, and 172 bp.

## Discussion

Corneal dystrophy is a group of diseases with autosomal dominant inheritance. Until now, corneal dystrophies failed to be clearly classified because of the variability in phenotypic expression of the diseases. A proposed corneal dystrophy classification system, which is identical or similar to those in the current nomenclature, is anatomically based with dystrophies classified according to the layer mainly involved, such as the epithelial and subepithelial, Bowman’s layer, stroma, Descemet’s membrane, and endothelium [16]. Clinical characteristics such as the depth of the cornea affected, the morphology of the deposits, and the histopathological features are also important for classifying different corneal dystrophies [17]. However, dystrophies with overlapping and atypical characteristics are still too similar to be distinguished from one another. A Chinese family with atypical RBCD was recently reported [14]. This family presented with a unique corneal dystrophy within the Bowman’s layer and the corneal stroma. However, no lattice was noted in the proband or other affected members, and the deposits were located in the mid-stroma of the cornea, which was different from the phenotypes previously reported in lattice corneal dystrophy patients with R124C mutation. Some corneal dystrophies affect multiple corneal layers and therefore cannot be classified as a single type based on morphologic criteria.

Phenotypically, the pedigree we documented here exhibited typical features of RBCD. The affected individuals presented with a gray-white geographic opacity in the anterior to mid-stroma of both eyes. In addition, geometric and round opacities in the subepithelial layers and anterior to mid-stroma were found in all of the affected family members. The clinical features, including recurrent erosion and gradually developing opacities of the Bowman’s layer, were consistent with the characteristic of RBCD [1,18] and to those found in the families previously described by Afshari et al. [10] and Aldave et al. [19]. In vivo confocal microscopy images were also consistent with the reported findings in RBCD [20].

*TGFBI* has been closely involved with the inherited corneal dystrophies, as mutations in this gene were identified in at least 5 types of corneal dystrophies, including granular corneal dystrophy (R555W), Avellino corneal dystrophy (R124H), lattice corneal dystrophy type I (R124C), Thiel-Behnke corneal dystrophy (R555L), and RBCD (R124L, G623D) [10,19]. Among those mutations reported, R124 appeared to be a “hot-spot” point mutation in *TGFBI* [21,22] as the R124 mutation has been detected in three types of corneal dystrophies, including Avellino corneal dystrophy, lattice corneal dystrophy type I, and RBCD. In previous reports, R124C is associated with lattice corneal dystrophy [15,21,23]. Interestingly, R124 in exon 4 of *TGFBI* is conserved among several species, including *Homo sapiens*, *Mus musculus* (R124), *Pan troglodytes* (R124), *Macaca mulatta* (R124), and *Rattus norvegicus* (R124), and is incompletely conserved in *Gallus gallus* (R117) and *Danio rerio* (R118). It strongly suggests that this residue is an important—functional and structural—site of the protein.

To date, *TGFBI* was the only gene found to be associated with RBCD. Here, we demonstrated an unusual R124C mutation in *TGFBI* associated with the RBCD, which was a mutation known to be responsible for corneal lattice dystrophy type I. Therefore, along with G623D and R124L, the R124C mutation in *TGFBI* is also found to be responsible for RBCD.

In 1996, Small et al. [24] suggested that Reis-Bücklers, lattice type 1, Avillino, and granular corneal dystrophies are all the same disease as they stem from the same gene products. Recently, it was suggested by the International Committee for Classification of Corneal Dystrophy that the following corneal dystrophies be named as *TGFBI* corneal dystrophies, including granular corneal dystrophy type 3 (RBCD), Thiel–Behnke corneal dystrophy (TBCD), classic lattice corneal dystrophy (LCD1), Granular corneal dystrophy, type 1 (classic; GCD1), and Granular corneal dystrophy, type 2 (granular-lattice; GCD2) [16]. Corneal dystrophies caused by mutations in *TGFBI* are characterized by abnormal extracellular deposits of mutated TGFBI protein in the corneal stroma. Most of TGFBI mutations so far reported are located in the fourth Fas1 domain with two mutational hot spots in R124 and R555. There is a strong correlation between phenotype-genotype in most corneal dystrophies caused by *TGFBI* mutations. The corneal dystrophy with R124L mutation usually has a worse prognosis, whereas the clinical manifestations of corneal dystrophies resulting from R555T or R555C mutations are usually mild. Other mutations such as P501T and N622K are related to various subtypes of lattice-like dystrophies. However, as dystrophies have a known common genetic basis, they may be classified into a single category, *TGFBI* dystrophy [16].
